# How predictable is genome evolution?

**DOI:** 10.7554/eLife.50784

**Published:** 2019-09-13

**Authors:** Matthew J Coathup, Owen G Osborne, Vincent Savolainen

**Affiliations:** 1 Department of Life Sciences, Silwood Park CampusImperial College LondonAscotUnited Kingdom; 2 School of Natural SciencesBangor UniversityBangorUnited Kingdom

**Keywords:** speciation, genetic divergence, transcriptome sequencing, eastern Asia-eastern North America floristic disjunction, genome evolution, positive selection, Other

## Abstract

Similar patterns of genomic divergence have been observed in the evolution of plant species separated by oceans.

**Related research article** Dong Y, Chen S, Cheng S, Zhou W, Ma Q, Chen Z, Fu CX, Liu X, Zhao YP, Soltis PS, Wong GKS, Soltis DE, Xiang J. 2019. Natural selection and repeated patterns of molecular evolution following allopatric divergence. *eLife* **8**:e45199. doi: 10.7554/eLife.45199

Understanding the formation of new species – a process called speciation – is a central challenge in evolutionary biology and genomics, but many questions remain (Arnegard et al., 2014; Riesch et al., 2017). In particular, are there certain patterns of genome evolution that are repeated? And, if there are, can we predict how genomes will change as new species emerge and diverge?

To date, most studies have focused on the genomes of pairs of closely related species living in close proximity (such as two species of whitefish living in the same set of lakes; Gagnaire et al., 2013), or on the genomes of different lineages within a single species (such as one species of stick insect living on two different host plants; Soria-Carrasco et al., 2014), and have predominantly investigated scenarios where there is some degree of gene flow between the different species or lineages (Wolf and Ellegren, 2017). However, it is thought that most new species emerge in geographically isolated populations and in the absence of gene flow. Now, in eLife, a team of researchers from the United States, China and Canada – including Yibo Dong (North Carolina State University) and Shichao Chen (University of Florida and Tongji University) as joint first authors – report how pairs of closely related flowering plants which live thousands of miles apart genetically diverged during evolution (Dong et al., 2019).

Dong et al. systematically collected and sequenced 20 pairs of closely related species, including 16 pairs that diverged between 2 and 10 million years ago as a result of the Eastern Asian–Eastern North American floristic disjunction (Figure 1). These pairs of species are the ideal system in which to study allopatric speciation (that is, speciation in geographically isolated populations).

**Figure 1.**
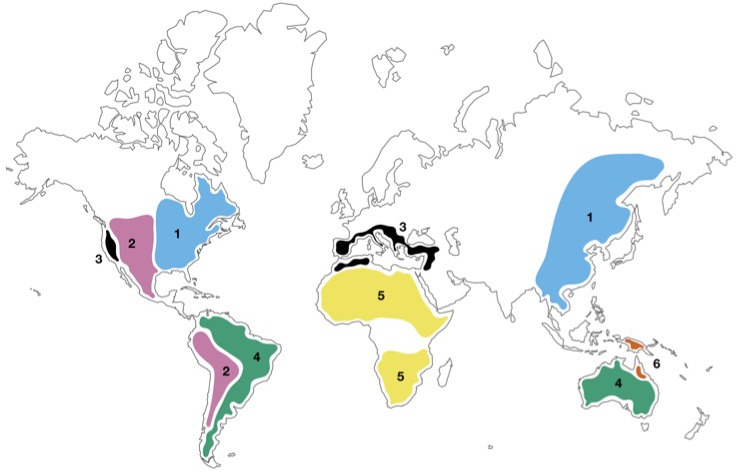
Geographical distribution of closely related plant species. Map showing floristic disjunctions – geographically separated regions than contain closely related plant species: 1) the Eastern Asian–Eastern North American floristic disjunction (blue; Dong et al., 2019); 2) the American Amphitropical floristic disjunction (pink; Raven, 1963); 3) the Madrean–Tethyan floristic disjunction (black; Wen and Ickert-Bond, 2009); 4) the Australia–South America floristic disjunction (green; van den Ende et al., 2017); 5) the Northern–Southern Africa floristic disjunction (yellow; Jürgens, 1997); 6) the disjunction between *Linospadix minor* in North-East Queensland and *L. albertisianus* in New Guinea (brown; Osborne et al., 2019).

Image credit: Matthew Coathup.

Dong et al. examined divergence in thousands of genes by measuring the rate at which nucleotide substitutions resulted in a change in the amino acid coded for (Ka), and the rate of nucleotide substitutions that did not result in such a change (Ks). The ratio Ka/Ks is a common index for identifying the selective pressure on a gene: values significantly above one indicate positive selection (i.e. an increase in the frequency of beneficial mutations), and values significantly below one indicate purifying selection (i.e. the removal of deleterious mutations). Despite investigating a diverse range of taxa, Dong et al. found that all species pairs experienced similar patterns of genomic divergence and selection, regardless of their ecologies and morphologies.

In fact, most of the genes measured showed little divergence and, intriguingly, the peak frequencies of Ks, Ka and Ka/Ks for each pair clustered within narrow ranges of small values. This is indicative of a common pattern in the relative numbers of genes at different levels of divergence during allopatric speciation, with only a small number of loci in the genome displaying high levels of divergence. Furthermore, by categorising the Ka/Ks ratios into six groups, Dong et al. found that in all pairs of species the relative number of genes under different selection pressures followed the same order. Moderate purifying selection was most common, followed by strong purifying selection, relaxed purifying selection, weak/moderate positive selection, putatively neutral selection and, finally, strong positive selection. Overall the patterns observed by Dong et al. suggest that it might be possible to make predictions about genome evolution.

While the study focused on the Eastern Asian–Eastern North American floristic disjunction, it is likely that these patterns of genome evolution are a common feature of allopatric speciation. Many other disjunctions exist around the world (Figure 1), and similar analyses of these would determine whether the findings of Dong et al. represent a common rule of genomic divergence. Indeed, recent work by some of the present authors and colleagues has shown that two pairs of palm species – one widely separated, the other not – have values of Ka, Ks and Ka/Ks similar to those reported by Dong et al. (Osborne et al., 2019). This supports the idea that these patterns may be common to genomic divergence in general, regardless of geography.
